# Split flow humidity generator equilibration and stability study

**DOI:** 10.1038/s41598-021-04073-2

**Published:** 2022-01-10

**Authors:** Justin M. Curtiss, Darren K. Emge

**Affiliations:** grid.420176.6 U.S. Army Combat Capabilities Development Command Chemical Biological Center, 5198 Blackhawk Road, Aberdeen Proving Ground, MD 21010 USA

**Keywords:** Hydrology, Chemical education, Process chemistry

## Abstract

Generation and control of humidity in a testing environment is crucial when evaluating a chemical vapor sensor as water vapor in the air can not only interfere with the sensor itself, but also react with a chemical analyte changing its composition. Upon constructing a split-flow humidity generator for chemical vapor sensor development, numerous issues were observed due to instability of the generated relative humidity level and drift of the humidity over time. By first fixing the initial relative humidity output of the system at 50%, we studied the effects of flowrate on stabilization time along with long term stability for extended testing events. It was found that the stabilization time can be upwards of 7 h, but can be maintained for greater than 90 h allowing for extended experiments. Once the stabilization time was known for 50% relative humidity output, additional studies at differing humidity levels and flowrates were performed to better characterize the system. At a relative humidity of 20% there was no time required to stabilize, but when increased to 80% this time increased to over 4 h. With this information we were better able to understand the generation process and characterize the humidity generation system, output stabilization and possible modifications to limit future testing issues.

## Introduction

Replicating realistic environmental conditions on a small scale is essential when testing and developing chemical vapor sensors. In particular, the presence of water vapor, or relative humidity (RH), in the air is a real world challenge in almost all scenarios and must be taken into consideration as it can have a dramatic impact on a sensor’s performance.

Ambient humidity can cause an instrument to produce a false alarm or fail to alarm (false negative) depending on the sensor type or algorithm. Moreover, it is also possible for the water vapor to interact with a target chemical vapor, analyte, through hydrolysis causing the chemical structure to change thus further complicating the problem^1–4^. Therefore, it is possible that under completely dry conditions, very low RH, a sensor would detect a chemical, but under humid conditions the sensor may not due to the presence of hydrolysis breakdown products or the influence of the ambient RH.

During testing and evaluation, humidity can be generated and controlled using numerous commercial or non-commercial systems, including the split-flow design utilized for the work detailed in this paper^5,6^. The split-flow system presented was designed and built in-house from commercially available components and its performance monitored using in-house programmed software and commercially available transducers. While the system was simple to operate and control, several issues were observed during initial use and testing. Constant adjustments to the humidity control valves were required to counteract the fluctuations and instability in the humidity output and levels within a test chamber. Due to these observed fluctuations, the ability to perform long term experiments would be questionable, as constant adjustments to the system would be required to compensate for the instability in RH levels. The time required for the system to reach a stable RH level, equilibration time, is also unknown. Because of the instability of the humidity generated using this type of system, further investigation of the problem was required to gain a better understanding of the problem in order to mitigate the issues outlined above.

While a split-flow design is an established method for generating humidity, the issues observed regarding stabilization time of a system and the resultant decrease in humidity are not well documented. Experiments and results documented herein shed light on an often overlooked or undocumented area of research that would likely be encountered by a user of this system type. This study only investigates one method of humidity generation, but the resulting information could be applied to other methods as they may be impacted by similar environmental and physical limitations.

## Principle of humidity

Humidity is the concentration of water vapor in the air, which has both a pressure and temperature dependency^7^. Expressing humidity is possible using a number of different measurements with RH being common. RH is the ratio of partial vapor pressure to equilibrium vapor pressure of water in the air at a specific temperature. The pressure dependency of humidity is related through Dalton’s law where the total exerted pressure (P_total_) is equal to the sum of the partial pressures of the gases, as shown through Eq. (1), with P_1_, P_2_, … P_n_ representing the partial pressures of the individual components of the system; water, air and chemical analyte are the components of interest in this work.

1 $${P}_{total}={P}_{1}+{P}_{2}+\dots +{P}_{n}.$$

Assuming the total pressure of a system remains constant at ambient atmosphere, as the output of the system is open to atmosphere and therefore will be pressurized, than as the partial pressure of one gas increases the other(s) must decrease, as related through Eq. (1), i.e., as humidity is added or increased in a system the effective concentration of the target chemical is decreased^8^. The temperature dependency of RH is represented by dew point that specifies the temperature at which the ambient air is saturated with water vapor. To increase the water content in the air, the temperature must be increased, in contrast if the temperature is decreased the water vapor will condense from the air, as defined by the relationship presented in Eq. (2), where Td is dew point temperature, RH is relative humidity, t is temperature and, the coefficients: A_1_ = 7.624, B_1_ = 243.04 °C^7^.

2 $$T_{d} = \frac{{B_{1} \left[ {\ln \left( {{\raise0.7ex\hbox{${RH}$} \!\mathord{\left/ {\vphantom {{RH} {100}}}\right.\kern-\nulldelimiterspace} \!\lower0.7ex\hbox{${100}$}}} \right) + {\raise0.7ex\hbox{${A_{1} t}$} \!\mathord{\left/ {\vphantom {{A_{1} t} {B_{1} + t}}}\right.\kern-\nulldelimiterspace} \!\lower0.7ex\hbox{${B_{1} + t}$}}} \right]}}{{A_{1} - \left[ {\ln \left( {{\raise0.7ex\hbox{${RH}$} \!\mathord{\left/ {\vphantom {{RH} {100}}}\right.\kern-\nulldelimiterspace} \!\lower0.7ex\hbox{${100}$}}} \right) + {\raise0.7ex\hbox{${A_{1} t}$} \!\mathord{\left/ {\vphantom {{A_{1} t} {B_{1} + t}}}\right.\kern-\nulldelimiterspace} \!\lower0.7ex\hbox{${B_{1} + t}$}}} \right]}}.$$

It should be noted that Eq. (2) provides only a good approximation of the dew point temperature as coefficients have errors associated with them as well as a temperature dependence, and methods to calculate RH, based on the Magnus formula, makes several assumptions. These assumptions increase the uncertainty in the measured or calculated value for the dew point temperature and RH, with a relative error of less than 0.4% over a temperature range of − 40 to 50 °C. For the work reported, all experiments took place at ambient conditions, or approximately 22 °C. In addition, an error of 0.4% is less than the error of the instrument used to measure the relative humidity of the system, discussed in “Description of apparatus” section. Understanding how the partial pressures of air, water vapor and chemical analyte in the system are affected by not only each other but also temperature allows for accurate control of each component yielding more reproducible results.

**Figure 1:**
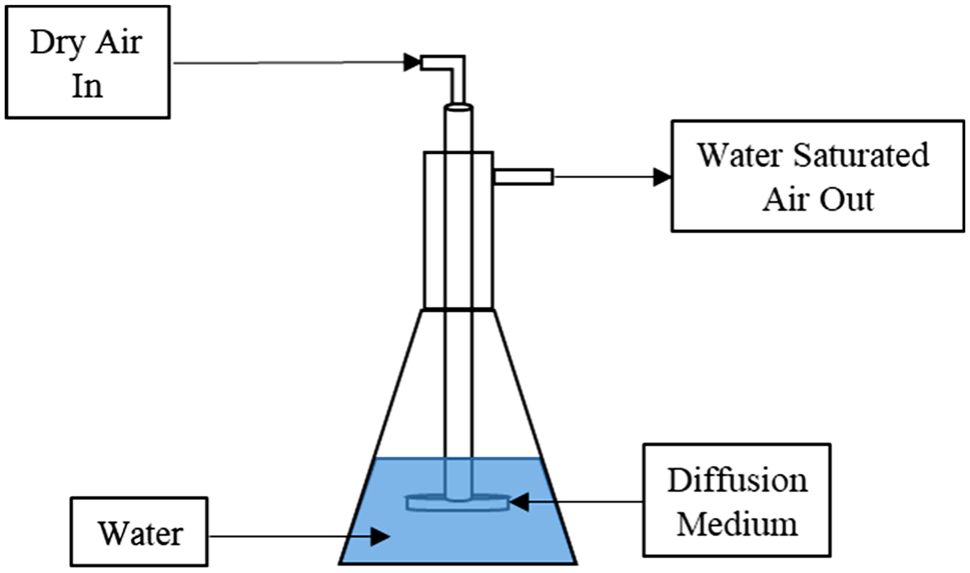
Conceptual water sparger design.

During test and evaluation of the humidity generator, a controlled humid air stream was generated through sub-surface aeration of a water reservoir using a sparger, as shown in Fig. 1, where stream of dry air is pushed through a diffusion medium under a column of water, saturating the air with water vapor. The size, shape, and porosity of the diffusion medium as well as column depth can all affect the aeration efficiency, water vapor transport, and thus humidity generation rate^9^. By fixing these variables, the relationship between air flowrate, RH generated and the equilibration time can be investigated.

## Description of apparatus

**Figure 2:**
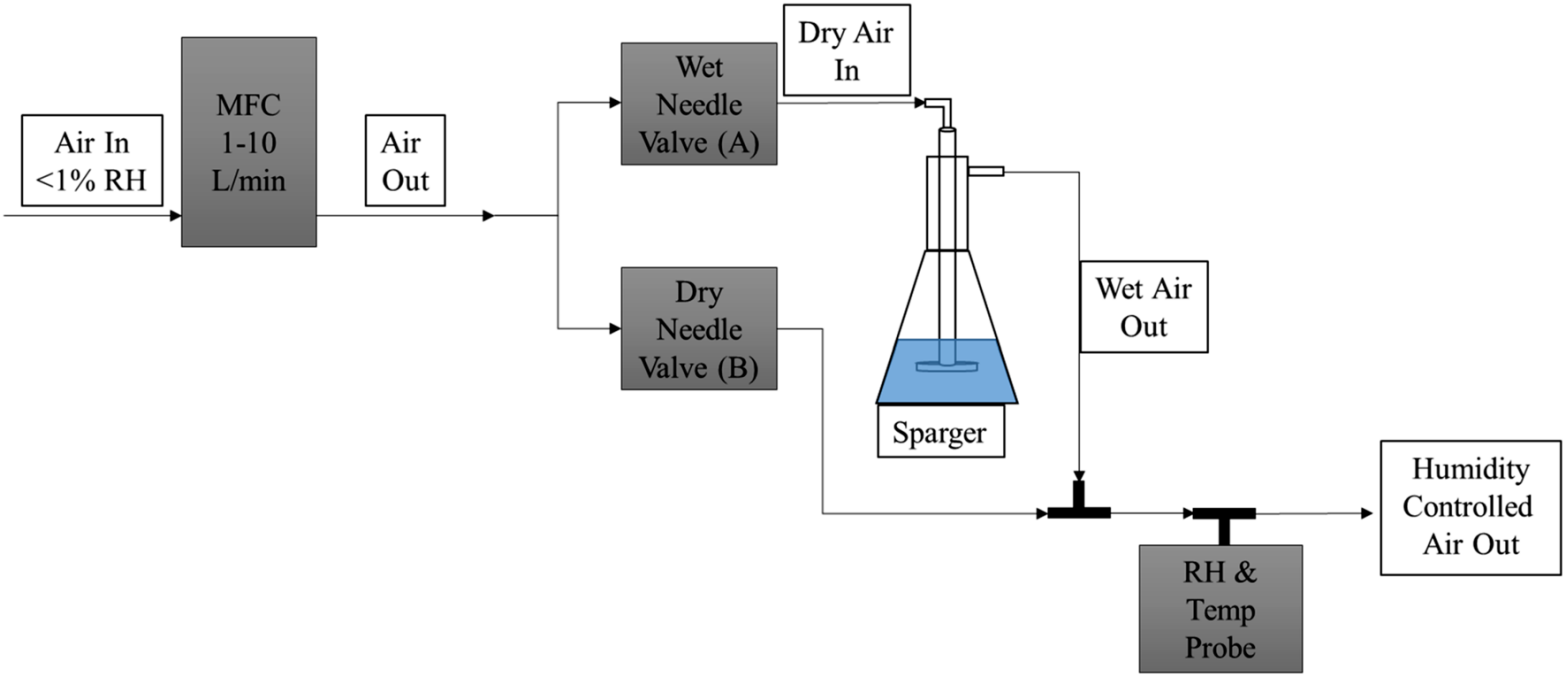
Humidity generator/controller schematic.

Many types of humidity generators and controller methods exist, they range from split flow, multi flow, to salt solution baths, and pressure systems^4,5,10^. The system described in this paper is of a split flow design constructed from commercially available components with the schematic shown in Fig. 2. A single compressed dry house air supply is split into two streams, one is dry air and the other is used to generate a ‘wet’ or water saturated stream. These two streams are combined to create a single controlled humidity stream. The ratio of dry to water saturated air is controlled by adjusting two needle valves, delineated as A and B, (Swagelok, SS-4L) placed after a splitting tee (Swagelok, SS-400-3) with the source dry air supplied by a single mass flow controller (MFC, Aalborg GFC 1–10 L/min, ± 1%). This configuration maintains a constant total air flow volume of the system. The outlet of valve A is routed to a 2 L Erlenmeyer style sparger (Chemglass, custom sized) flowing air through a glass aeration disc in a volume of water creating a water saturated air stream. The dry and wet streams are recombined with a tee fitting, creating a single stream of humid air that can be delivered to a test chamber. An inline hygrothermometer (Omega, HX200) measures and displays the temperature and RH in real-time with an accuracy of ± 0.5 °C and ± 1% RH, allowing the humidity level to be adjusted with valves A and B to the desired RH; the temperature and RH of the air output can change downstream due to temperature changes outside transfer lines, as described by Eq. (2), and should be monitored for greater accuracy. Air flow rates through the MFC, temperature and RH, from the inline hygrothermometer are recorded with LabVIEW based software.

A split flow design offers several advantages over other common types of humidity generators and controllers^4^. Construction of the system is simple, can be built in-house using commercially available components, with the exception of the sparger, which was custom sized, but there are commercial alternatives available (Mott micro sparger tips). The use of a single MFC maintains the total flow volume of the system rather than multiple MFCs used for a multi flow design, removing a source of error in the total flow volume and the need to adjust and control multiple MFCs. When mixing the humid air stream with a chemical analyte supplied by a separate MFC, calculations are needed to determine the mixing ratios due to the additional supply of analyte stream. This is again simplified by using a single MFC as opposed to multiple MFCs to control the dry and wet streams separately. Safety is also a consideration for the generation system, where use of a split flow design maintains the system at near ambient pressure unlike a two pressure system that generates a pressurized saturated stream that is mixed with the dry stream in an expansion chamber. For this effort we did not address temperature management and instead operated at ambient laboratory temperatures of 21 ± 1 °C. Note that a temperature change of 1 °C would result in a RH change of approximately 3% which were observed, but did not greatly impact the experiments. In summary, a split-flow design provides a simple solution to humidity generation and control using commercially available components.

## Methods and experiments

Initial experiments were conducted to determine if the system could produce a steady stream of humid air, as well as the time required to reach a steady state, or equilibrium. An initial target RH of 50% was chosen, a common setting based on past experimental needs, with a flowrates in the range of 1 to 10 L/min. Prior to the start of the air flow, the wet valve was closed and dry valve was fully opened. The sparger was filled with deionized water to a predetermined level, and the desired air flowrate was set in the MFC. The air flow was started and the system was allowed to run until the reported RH level was below 2% to remove any condensed water remaining in in the lines from previous experiments. After the system was cleared of any residual moisture, the level was visually monitored and needle valves adjusted until the 50% RH target was reached and remained unchanged for approximately 1 min. The system was then allowed to run for a minimum of 12 h with the temperature and RH values recorded every second by the LabVIEW software.

Experiments were conducted for flowrates of 1, 2, 3, 4, 4.5, 5, 7, 8.5 and 10 L/min all initiated at the starting RH of 50%. The flowrates and range were chosen as they are common measurement rates for commercially available handheld gas sensors. Equilibration was defined as a change in RH of ± 2% to determine the equilibration time. This value was selected as this is a commonly observed fluctuation outdoors^11^. This level of fluctuation is within tolerances of many commercially available environmental chambers.

After establishing an equilibration time for the system over a range of air flowrates, the observed decrease in RH over time was investigated. Repeating the initial steps from the previous experiments, the starting RH was set to about 57%, above the target RH of 50%, at a flowrate of 5 L/min and allowed to run for 12 h. The temperature and RH were monitored and recorded continuously, with the goal for the system to equilibrate at the target RH of 50%.

To determine the effect of different starting RH values on time to equilibration and stability, a series of experiments were conducted where the starting RH was set to 20, 50 or 80%, with flowrates of 2, 5, 7, 8.5 or 10 L/min. These experiments were conducted in triplicate, with the system to running for 12+ h per replicate. The goal was to establish the relationship between, RH, flowrate and equilibration time.

## Results and discussion

Initial equilibration time experiment results with a target RH of 50% and flowrates ranging from 1 to 10 L/min are shown below in Table 1.

**Table 1:** Initial equilibration—50% RH.

Flowrate (L/min)	Target RH (%)	Starting RH (%)	Equilibrium RH (%)	Δ RH^1^	Equilibrium time (min)
1	50	50	50	0	0
1	50	50	50	0	0
2	50	50	48	2	0
2	50	50	49	1	0
3	50	50	47	3	420
4	50	50	47	3	420
4.5	50	50	45	5	480
5	50	51	44	**7**	300
5	50	50	43	**7**	210
5	50	51	46	5	240
7	50	50	45	5	500
7	50	50	45	5	500
7	50	51	47	4	350
8.5	50	50	44	6	350
8.5	50	50	45	5	500
8.5	50	50	45	5	500
10	50	51	44	**7**	400
10	50	50	43	**7**	375
10	50	50	43	**7**	420

^1^Bold values denote greatest change from starting to equilibrium RH.

At a flowrate of 1 and 2 L/min there was no significant change in RH over the duration of the experiment and thus no time for the humidity generator to equilibrate is required. As the flowrate of air increased above 3 L/min, there was continuous decrease from initial RH and the equilibrium RH with the greatest decrease around 7% which was observed at flowrates of 5 and 10 /min. The time to equilibrate ranged from about 210 to 500 min (3.5 to 8 h) over the flowrate range of 3–10 L/min. No obvious trends in the equilibration time or equilibrium humidity were observed, such as a linear decrease in humidity with flowrate. The equilibration time varied by as much as 150 min within the same flowrate experiments. There were increasing and decrease equilibration times observed with increase flow rate, e.g. at 5 L/min the equilibration time was 210–300 min, but at both and 7 L/min the equilibration time was greater. The volume of the sparger may have played a role in this as it has volume of only 2 L, with approximately 1.5 L of water. At 10 L per minute, five times the volume of air is being pushed through the sparger than its volume and more than six times the volume of water it contains. Later experiments will show that at lower humidity levels and thus lower air flow rates through the sparger, no equilibration time is required.

For higher flowrates the volume of water in the sparger decreased slightly over longer test periods of up to 96 h, however no noticeable change in RH was observed due to the water volume changing. Therefore, longer term, multi-day experiments could be performed with minimal concern of effects due to the sparger volume decrease.

**Figure 3:**
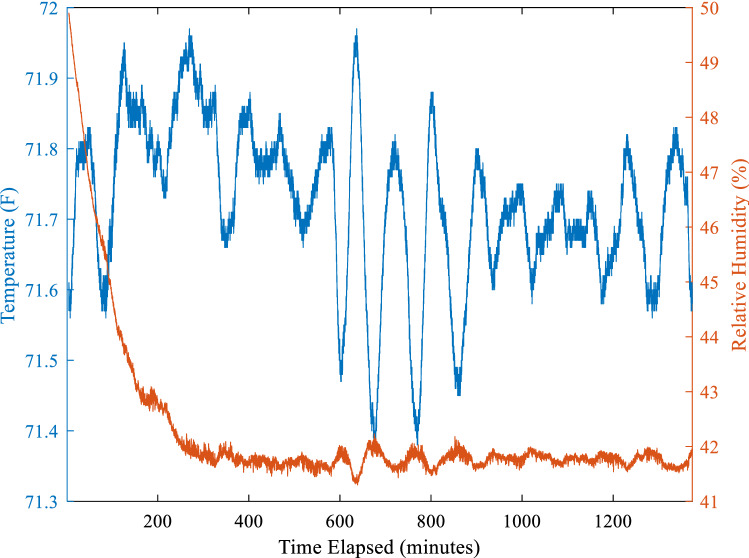
Example data readout of temperature and RH over 22 h, 10 L/min at 50% RH.

Fluctuations in RH were observed to be inversely proportional to the temperature, as expected due to the dew point temperature relation described Eq. (2), and is shown in Fig. 3^12^. The overall temperature change was less than 1 °C, presumably from variations in laboratory air temperature. This resulted in a RH fluctuation of less than 1% after equilibrium, which is within the tolerance defined above. While there is no direct evidence that evaporative cooling within the sparger occurred, it is possible that the drop in RH until equilibrium is reached is due to this effect. Additional temperature probes could be placed within the sparger air space and along the air flow path to determine this was in fact the source of the observed variation.

**Figure 4:**
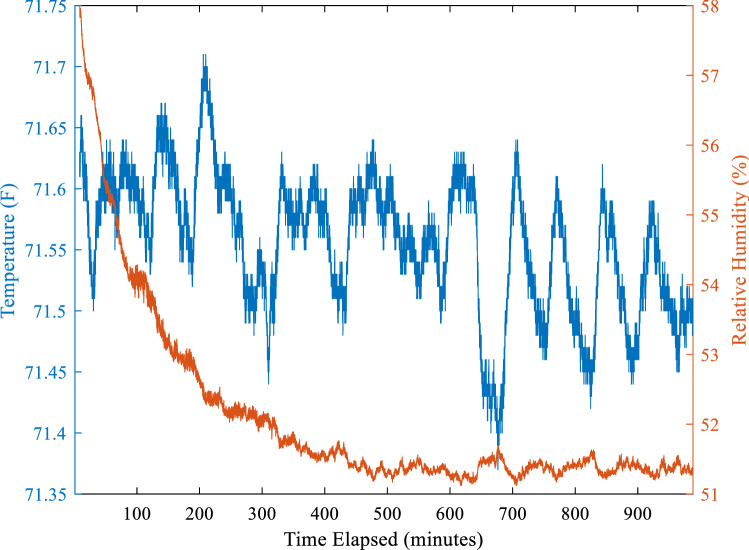
Example compensation for humidity drop starting at 58% and decreasing to 51% over 8 h with stability for additional 8 h.

To counteract the observed decrease in RH during equilibration, compensation could be made by overshooting the starting RH level and allowing the system to equilibrate to the target RH. With a target RH of 50% and an observed decrease of 7% in 5 experiments, denoted by the bold text of Table 1, the starting RH was adjusted to be about 58%. By adjusting for the decrease in RH during equilibration the desired set point the target RH of about 50% can be achieved. In this experiment, the system was set to 5 L/min with a starting RH of about 58% and allowed to equilibrate over eight hours. The equilibrium RH level decreased from the set point of about 58% to the target of about 51%, with an example shown in Fig. 4. This suggests compensation for some drop in RH is possible in order to achieve a target RH and reach stability without the need for additional adjustments. The decrease in equilibrium RH is not consistent across all flow rates, therefore the starting RH level would need to be adjusted differently based on the flow rate.

Several experiments were allowed to run for up to 96 h to assess the long term stability of the generation system. Such long experimental runs support multi day testing and demonstrate that the humidity generation can be started the day before testing in order to allow the system equilibrate. At a flowrate of 2, 5, and 10 L/min, generation of humidity was stable for 86, 91, and 96 h, respectively, though longer generation period is possible as the sparger water volume only slightly decreased. As previously discussed, decrease in the sparger water volume did not have a noticeable effect on humidity generation or stability, at < 1% RH change, though this may not hold true on a larger scale, such as a pond^9^. While equilibration time was observed to be up to 8 h, the ability to start the system the prior day and allow it to come to equilibrium over night with little effect on performance negates some concerns associated with required long equilibration periods.

**Figure 5:**
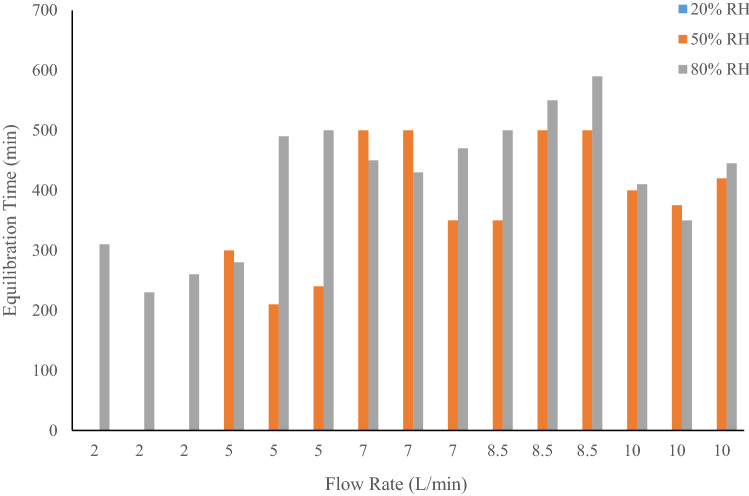
Equilibration time of system versus flow rate and starting humidity.

To study equilibration time and stability of RH environmental conditions other than 50%, experiments were conducted at 20 and 80% RH. Flowrates of 2, 5, 7, 8.5 and 10 L/min were used and the results are shown in Table 2 and displayed in Fig. 5.

**Table 2:** RH vs. flowrate results.

Flowrate (L/min)	Target RH (%)	Starting RH (%)	Equilibrium RH (%)	Equilibrium time (min)
2	20	20	20, 20, 20	0, 0, 0
5	20	20	21, 21, 21	0, 0, 0
10	20	20	18, 19, 18	0, 0, 0
2	50	50	48, 51, 49	0, 0, 0
5	50	51	44, 43, 46	300, 210, 240
7	50	50	45, 45, 47	500, 500, 350
8.5	50	50	44, 44, 45	350, 500, 500
10	50	50	44, 43, 43	400, 375, 420
2	80	80	76, 77, 76	310, 230, 260
5	80	80	70, 69, 69	280, 490, 500
7	80	80	67, 65, 64	450, 430, 470
8.5	80	80	64, 66, 69	500, 550, 590
10	80	79	60, 62, 62	410, 350, 445

For all of the experiments ran with a starting RH of 20%, there was no change observed in the RH through the course of the experiments, and therefore there was no equilibration time required. Unlike the experiments ran with a starting RH of 50% where at the lowest flow rates of 2 L/min there was no change in the RH, when the starting RH was increased to 80%, an equilibration time of at least 210 min was required. As the flow rate increased, so did the equilibration time, but for both the 50% and 80% start RH experiments at the highest flow rate of 10 L/min the equilibration time did decrease below the maximum observed value. The stabilization humidity level decreased with a range of 5 to 20%, with a greater decrease at the 80% RH starting point and higher flow rates of 8.5 and 10 L/min.

For higher flowrates and humidity levels the lack of aerator surface area could limit the interaction between the air and water preventing enough water content in the air thus resulting in greater decrease in RH level when stabilization is achieved. Aeration efficiency can be increased by increasing the aerator surface area, decreasing pore size to create smaller, more abundant bubbles, and increasing the water column length of the sparger. These changes increase travel path of air or number of bubbles allowing for more interaction between the water and air, creating more water saturated air^9^. This is supported by the results, comparing the lower 20% RH setting with the higher 80% RH, where at the lower setting there is no equilibration time, but at a higher humidity the sparger has to equilibrate due to the high air flow to create the more highly saturated water vapor. Another option to increase the saturated vapor pressure would be to increase the water temperature, however, this would require the rest of the system to be conditioned to avoid condensation. Ultimately, as the flowrate or initial RH setting increases, the stabilized RH drop also increases.

Further experiments are needed to determine if the decrease in RH levels at stabilization can be compensated for at high flowrates and RH levels. The highest observed RH after stabilization with the system during initial testing was 81% at 5 L/min with the wet valve fully open and dry valve closed. Overall, lower and higher humidity levels are achievable and sustainable using the split flow system with the knowledge that at RH levels of 80%, there is a considerable decrease once equilibrium is reached.

## Conclusions

A split flow humidity generator and controller provides a simple method to create a controlled humidity stream for chemical vapor sensor testing. Initial RH instability in the system prompted further investigation into equilibration and the factors that affect the stability. Starting with a target of 50% RH, the time to achieve stability across a range of 1–10 L/min flowrate was tested and it was determined that as the flow went beyond 3 L/min stabilization could take several hours, though once reached, remained stable for over 90 h. This opens up the possibility to perform longer term, multiday sensor evaluations. One complication to the equilibration process is a drop in the stabilized RH, however this decrease can be compensated for as shown experimentally. Similar trends were observed with a starting RH of 20 and 80%, where stabilization took upwards of 8 h with a decrease from the starting RH level, but remained stable for several days after. Future work would look into different types of aeration media that may more efficiently generate saturated water vapor at higher air flowrates as well as automating the needle valves to adjust for humidity drift.

## References

[1] Fonollosa J, Solórzano A, Marco S (2018). Chemical sensor systems and associated algorithms for fire detection: A review. Sensors.

[2] Seeley JA, Richardson JM (2007). Early warning chemical sensing. Lincoln Lab. J..

[3] Murray GM (2013). Detection and screening of chemicals related to the chemical weapons convention. Encycl. Anal. Chem..

[4] Ling-Hsi Chen CC (2018). Uncertainly analysis of two types of humidity sensors by a humidity generator with a divided-flow system. Sensors.

[5] Hari Krishan BS (2005). Mixed flow relative humidity generator. MAPAN J. Metrol. Soc. India.

[6] Schellenberg R (2001). How hard could that be? Practical humidity calibration experiences. Int. J. Meteorol..

[7] Lawrence MG (2005). The relationship between relative humidity and dew point temperature in moist air. Bull. Am. Meteorol. Soc..

[8] Silberberg MS (2009). Chemistry: The Molecular Nature of Matter and Change.

[9] Al-Ahmady KK (2006). Analysis of oxygen transfer performance of sub-surface aeration systems. Int. J. Environ. Res. Public Health.

[10] Nguyen JL, Dockery DW (2016). Daily indoor-to-outdoor temperature and humidity relationships: A sample across seasons and diverse climatic regions. Int. J. Biometerol..

[11] Milosevic N, Stepanic N, Babic M (2012). A relative humidity calibration from 5 °C to 45 °C in a mixed flow humidity generator. Therm. Sci..

[12] Takahashi C, Kitano H (1996). Calibration method of flow meters for a divided flow humidity generator. Sens. Actuators B Chem..

